# Bayesian networks identify determinants of outcomes following cardiac surgery in a UK population

**DOI:** 10.1186/s12872-023-03100-6

**Published:** 2023-02-06

**Authors:** Khurum Mazhar, Saifullah Mohamed, Akshay J. Patel, Sarah Berger Veith, Giles Roberts, Richard Warwick, Lognathen Balacumaraswami, Qamar Abid, Marko Raseta

**Affiliations:** 1 Royal Stoke University Hospital, Stoke on Trent, UK; 2 Faculty of Medicine Carl Gustav Carus, TU Dresden, Dresden, Germany; 3 Institute of Immunology and Immunotherapy, University of Birmingham, Edgbaston, Birmingham, B15 2TT UK

**Keywords:** Bayesian network, Risk stratification, EuroSCORE, Cardiac surgery, Outcomes

## Abstract

**Background:**

Traditional risk stratification tools do not describe the complex principle determinant relationships that exist amongst pre-operative and peri-operative factors and their influence on cardiac surgical outcomes. This paper reports on the use of Bayesian networks to investigate such outcomes.

**Methods:**

Data were prospectively collected from 4776 adult patients undergoing cardiac surgery at a single UK institute between April 2012 and May 2019. Machine learning techniques were used to construct Bayesian networks for four key short-term outcomes including death, stroke and renal failure.

**Results:**

Duration of operation was the most important determinant of death irrespective of EuroSCORE. Duration of cardiopulmonary bypass was the most important determinant of re-operation for bleeding. EuroSCORE was predictive of new renal replacement therapy but not mortality.

**Conclusions:**

Machine-learning algorithms have allowed us to analyse the significance of dynamic processes that occur between pre-operative and peri-operative elements. Length of procedure and duration of cardiopulmonary bypass predicted mortality and morbidity in patients undergoing cardiac surgery in the UK. Bayesian networks can be used to explore potential principle determinant mechanisms underlying outcomes and be used to help develop future risk models.

**Supplementary Information:**

The online version contains supplementary material available at 10.1186/s12872-023-03100-6.

## Background

Risk stratification models have been commonly used for objective comparison of treatment outcomes between different institutions. In recent years, they have been used more broadly to inform patients and health care professionals of peri-operative risk and direct resource allocation of healthcare [1]. The focus of risk modelling in cardiac surgery has largely been on the ability to predict short-term mortality. Consequently, major morbidity remains unaccounted for in most risk scores. Mortality prediction alone is insufficient in determining surgical outcomes [2] which include complications such as stroke, acute renal failure, major bleeding requiring repeat surgical intervention as well as Deep Sternal Wound Infection and use of resources such as length of stay and transfusion requirements. The Society of Thoracic Surgeons (STS) risk score does identify several morbidity outcomes but has not been validated in a UK population for this purpose [3]. Moreover, neither the European System for Cardiac Operative Risk Evaluation (EuroSCORE) nor the STS risk score include intra-operative factors such as duration of operation, cardiopulmonary bypass (CPB) nor aortic cross clamp times, all of which have been linked to increased mortality and morbidity rates [4–6].

Most risk stratification tools and previous studies investigating the surgical outcomes have analysed the outcomes of adult cardiac surgery using survival analysis or methods of regression analysis, including use of a propensity score for adjustment of confounders. However, such methods do not assess how patient demographics, co-morbidities, length of operation, severity of co-morbidities, clinical history, or type of operation relate to each other in the principle determinant process leading to a potential difference in outcome. In this paper, we build a Bayesian network for each of 4 key health outcomes; Death, New haemofiltration, New post-operative neurological deficit, and Return to theatre for thoracic bleeding or tamponade. We sought to understand whether machine learning can help identify significant variables associated with mortality and morbidity following adult cardiac surgery not accounted for in traditional risk modeling. Our purpose in this study was not to build a new predictive model but investigate for proof of concept whether Bayesian networks could be used to determine short term outcomes for adult cardiac surgery.

## Methods

### Data

Data were collected from all adult patients who underwent cardiac surgery at the University Hospital of North Midlands NHS Trust between April 2012 and May 2019 (inclusive). Thoracic aortic and any ‘emergency’/‘salvage’ procedures data were excluded from the initial search of the database. We were interested in four primary outcome variables observed within 30 days of the operation: (1) Death, (2) New haemofiltration/dialysis (HF). (3) New post-operative neurological deficit, and (4) Return to theatre for thoracic bleeding or tamponade. Outcomes which are defined as ‘subjective processes of care/resource use’ such as: use of post operative inotropic/mechanical ventricular support, post-operative red blood cell transfusion and length of post operative stay are provided in the Additional file 1. Definitions of ‘elective’/‘urgent’ and the above outcomes are those specified by the National Institute for Cardiovascular Outcomes Research (NICOR) [7]. Pre-operative variables are those specified by the EuroSCORE (ES) [8]. ES was calculated via the Society for Cardiothoracic Surgery in Great Britain and Ireland (SCTS). Duration of operation (DOO) was defined as the length of time taken from ‘knife to skin’ until application of the final wound dressings. There were 182 patient records with inconsistencies or missing values in the original data set and so data from these patients were excluded from the study.

### Statistical methodology

We used the machinery of Bayesian networks to identify the smallest subset of variables that could provide the necessary information for prediction of the outcomes of interest consistent with known, or possibly newly discovered logically consistent principle determinant relationships between the variables. Classical statistics methodology is inappropriate for this purpose [9]. A Bayesian network is a graphical model used to estimate and visualise the inter-dependencies between variables whose distribution is fully specified via associated Markov blankets [10] which were painted in red for all adverse outcomes of interest for clarity of exposition. Bayesian networks are defined for categorical data and thus all continuous variables must be discretised which was achieved via *k*-means clustering algorithm [11] in the absence of scientific consensus regarding the universality of human-specified cut-offs. The search space for the network-learning algorithm was reduced by mandating that future events cannot be a principal determinant of past observations [12]. This was achieved by assigning temporal values to all nodes. The analysis was undertaken using the BayesiaLab software [http://www.bayesia.com]. Continuous data were summarized via means and standard deviations in case the data followed the Gaussian distribution and by medians and interquartile ranges (IQR) otherwise. Normality was tested by means of Shapiro–Wilk test while the independence between variables was tested by means of Kendal rank correlation test. Significance was accepted at the 0.05 level. Further specification of the fundamental properties of Bayesian networks, details of discretization cut-offs, assignment of temporal values and network learning is presented in the Additional file 1. All statistical analysis was done in ‘R ‘statistical software tool [R Core Team, 2018].

## Results

The characteristics of the 4776 patients included in the study are shown in Table 1. 46.7% had an urgent procedure with 7.9% of patients requiring pre-operative nitrate/heparin use and only 0.2% (n = 11) requiring pre-operative inotropic support. The vast majority of patients had isolated Coronary Artery Bypass Grafting (CABG), with 2.4% of patients requiring CPB for procedures other than CABG ± valve procedures, such as excision of atrial myxoma or transvenous pacing wire removal. ES did not follow a Gaussian distribution (Shapiro–Wilk test, *p* < 0.001). Its mean was 2.3; median was 1.40 with IQR (0.60–3).

**Table 1:** Patient characteristics of our sample population and classification of operations performed

Age (mean years)	67.8 (SD 10.3)
Female	1141 (24.0%)
Diabetes	1205 (25.2%)
Previous PCI	564 (11.8%)
Previous cardiac surgery	106 (2.2%)
Pre-operative IABP	92 (1.9%)
Pre-operative inotrope	11 (0.2%)
Pre-operative intravenous nitrate/heparin	377 (7.9%)
Hypertension	3426 (71.7%)
History of smoking	2970 (62.2%)
History of neurological dysfunction	396 (8.3%)
History of severe renal impairment/dialysis	51 (1.1%)
*LV ejection fraction*	
Good	3086 (64.6%)
Moderate	1428 (29.9%)
Poor	262 (5.5%)
Elective	2546 (53.3%)
Urgent	2230 (46.7%)
*Procedure type*	
Isolated CABG	2705 (56.6%)
Isolated valve(s)	1096 (22.9%)
CABG + valve(s)	629 (13.2%)
CABG + other	48 (1.0%)
Valve(s) + other	185 (3.9%)
Other	113 (2.4%)
SCTS EuroSCORE (mean)	2.3 (SD 2.7; Median 1.4; IQR 0.6–3.0)

Total population = 4776 patients. *SD* standard deviation, *PCI* Percutaneous Intervention, *IABP* Intra-Aortic Balloon Pump, *LV* Left Ventricle, *CABG* Coronary Artery Bypass Graft

Table 2 summarises overall outcomes and shows mean DOO was 242 min with CPB mean time of 124.2 min. In general, CPB increased in proportion to the length of DOO. We observed that CPB and DOO did not follow a bivariate normal distribution (Shapiro–Wilk test, *p* < 0.001). Kendall rank correlation test demonstrated significant correlation (*p* < 0.01, τ = 0.56) between CPB and DOO.

**Table 2:** Operative data and in-patient post-operative outcomes

		Range	Median; Interquartile Range
CPB usage	4321 (90.5%)		
OPCAB cases	449 (9.4%)		
Mean duration of all operations (minutes)	242.0 (SD 96.6)	30–1440	224; 180–279
Mean duration of OPCAB	250.1 (SD 74.3)	44–858	240; 204–278
Mean CPB time (minutes)	124.2 (SD 68.7)	20–681	108; 78–151
Mean AXC time (minutes)	85.6 (SD 56.9)	0–466	72; 44–111
New atrial fibrillation	1358 (28.4%)		
New permanent pacemaker	38 (0.8%)		
Use of post-operative IABP	64 (1.3%)		
*New neurological deficit*			
CVA/TIA	46 (1.0%)		
Other	3 (0.1%)		
New HF	117 (2.4%)		
Return to theatre for bleeding/tamponade	238 (5.0%)		
RBC transfusion	1803 (37.8%)		
DSWI	44 (0.9%)		
Postoperative stay (days)	9.2 (SD 8.5)	0–169	7; 5–10
Mortality	78 (1.6%)		
CPB usage	4321 (90.5%)		
OPCAB cases	449 (9.4%)		
Mean duration of all procedures (minutes)	242.0 (SD 96.6)	30–1440	224; 180–279
Mean duration of OPCAB	250.1 (SD 74.3)	44–858	240; 204–278
Mean CPB time (minutes)	124.2 (SD 68.7)	20–681	108; 78–151
Mean AXC time (minutes)	85.6 (SD 56.9)	0–466	72; 44–111
New atrial fibrillation	1358 (28.4%)		
New permanent pacemaker	38 (0.8%)		
Use of post-operative IABP	64 (1.3%)		
*New neurological deficit*			
CVA/TIA	46 (1.0%)		
Other	3 (0.1%)		
New RRT	117 (2.4%)		
Return to theatre for bleeding/tamponade	238 (5.0%)		
RBC transfusion	1803 (37.8%)		
DSWI	44 (0.9%)		
Postoperative stay (days)	9.2 (SD 8.5)	0–169	7; 5–10
Mortality	78 (1.6%)		

**Fig. 1:**
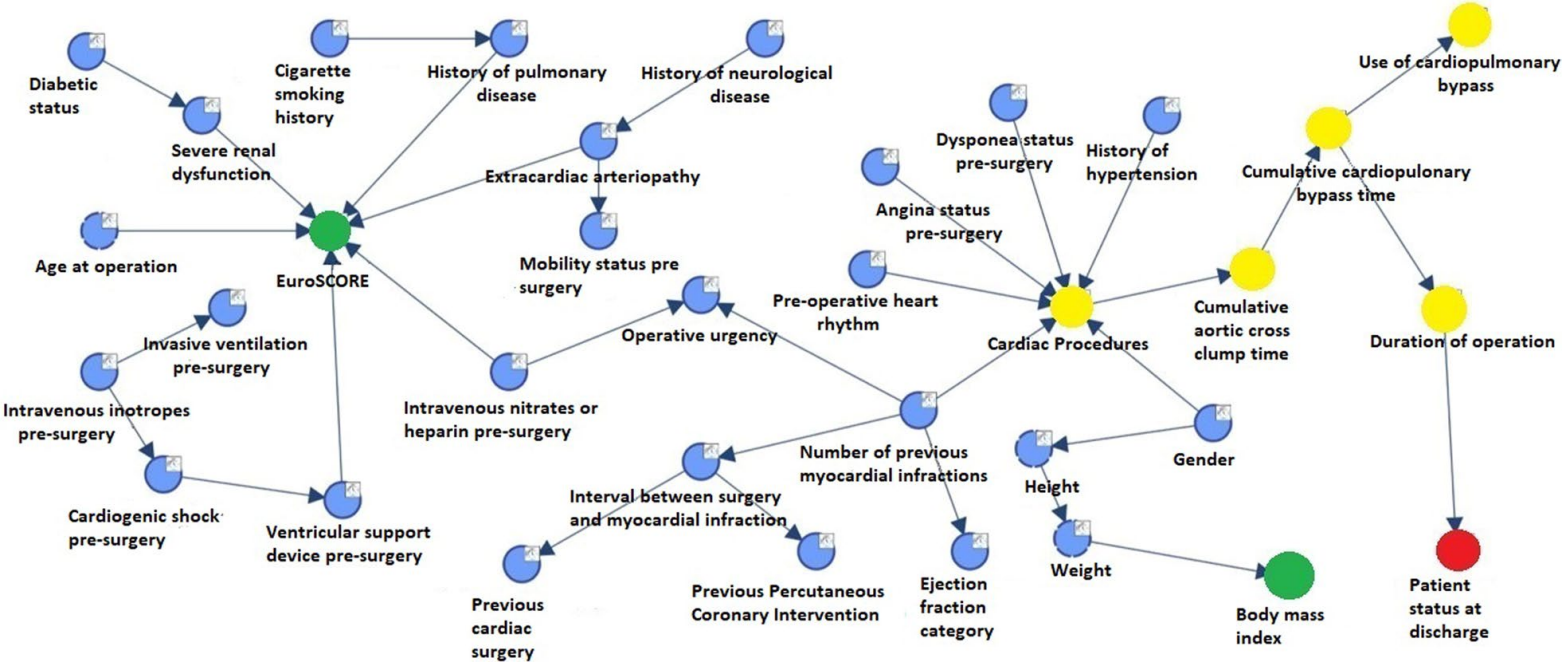
Bayesian network graph with MB for survival (‘status at discharge’)

*CPB* Cardiopulmonary Bypass, *OPCAB* Off-pump Coronary Artery Bypass Graft, *AXC* Aortic Cross Clamp, *IABP* Intra Aortic Balloon Pump, *CVA* Cerebrovascular Accident, *TIA* Transient Ischaemic Attack, *HF* Haemofiltration/dialysis, *RBC* Red Blood Cell, *DSWI* Deep Sternal Wound Infection,

The MB of the variable ‘Survival’ (patient status at discharge) (Fig. 1) consists only of the variable ‘Duration of Operation’. Hence, ‘survival’ is independent of all other variables given ‘Duration of Operation’. It follows that, in-patient mortality is only dependent upon the length of operation, which was a child of the CPB time and so indirectly influenced the mortality. ES was not found to predict survival in our study. Less than 1% of patients whose duration of operation was < 245 min died. Conversely, death rate for those patients whose operation lasted more than 421 min was 11.8%.

**Fig. 2:**
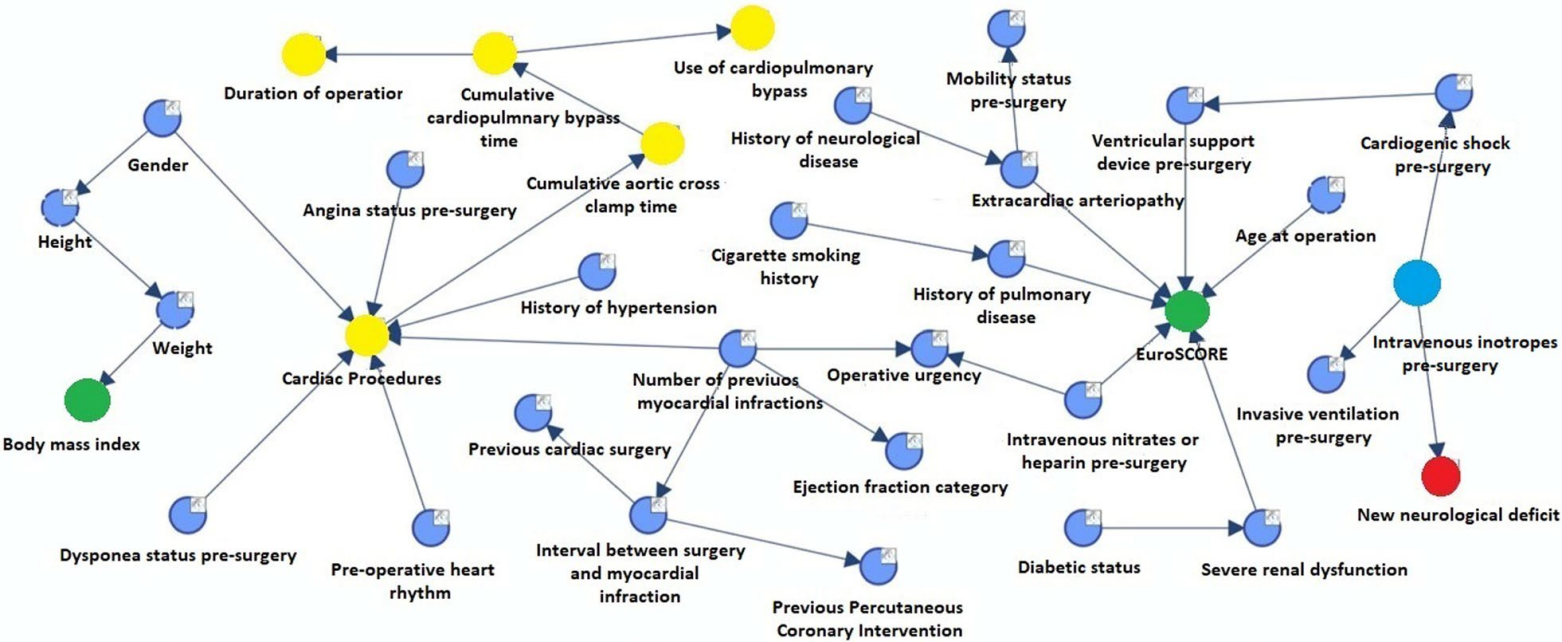
Bayesian network graph with MB for the variable ‘new neurological deficit’

**Fig. 3:**
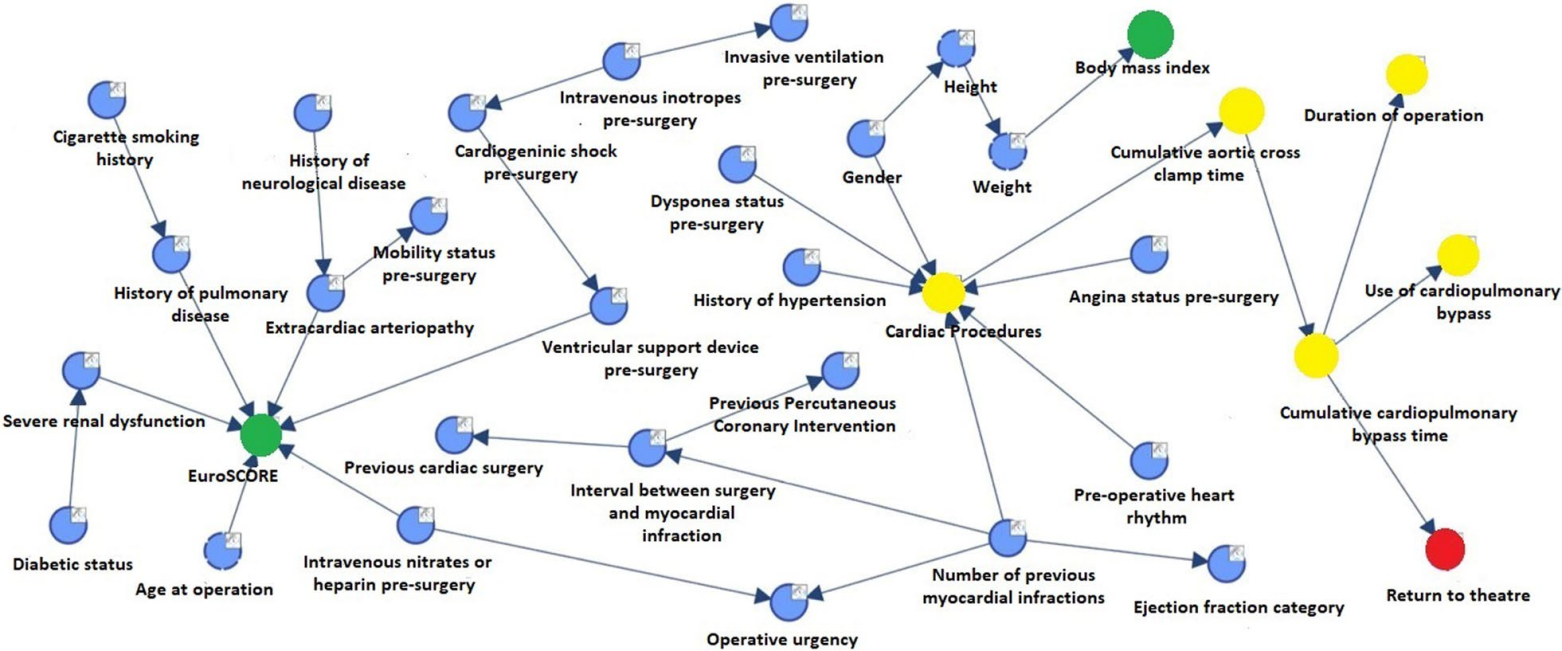
Bayesian network graph with MB for the variable ‘return to theatre for bleeding/tamponade’

New neurological deficits (Fig. 2) were dependent on pre-surgery inotrope usage irrespective of previous neurological history, the presence of peripheral vascular disease or type of procedure. Twenty percent of patients requiring pre-operative inotropic support had a stroke or TIA, however this figure was < 1% for patients requiring no support. The MB for ‘Return to theatre for bleeding/tamponade’ (Fig. 3) was ‘cumulative CPB time’ alone. Thus, in particular, ‘Return to theatre for bleeding/tamponade’ was independent of age or nature of procedure.

**Fig. 4:**
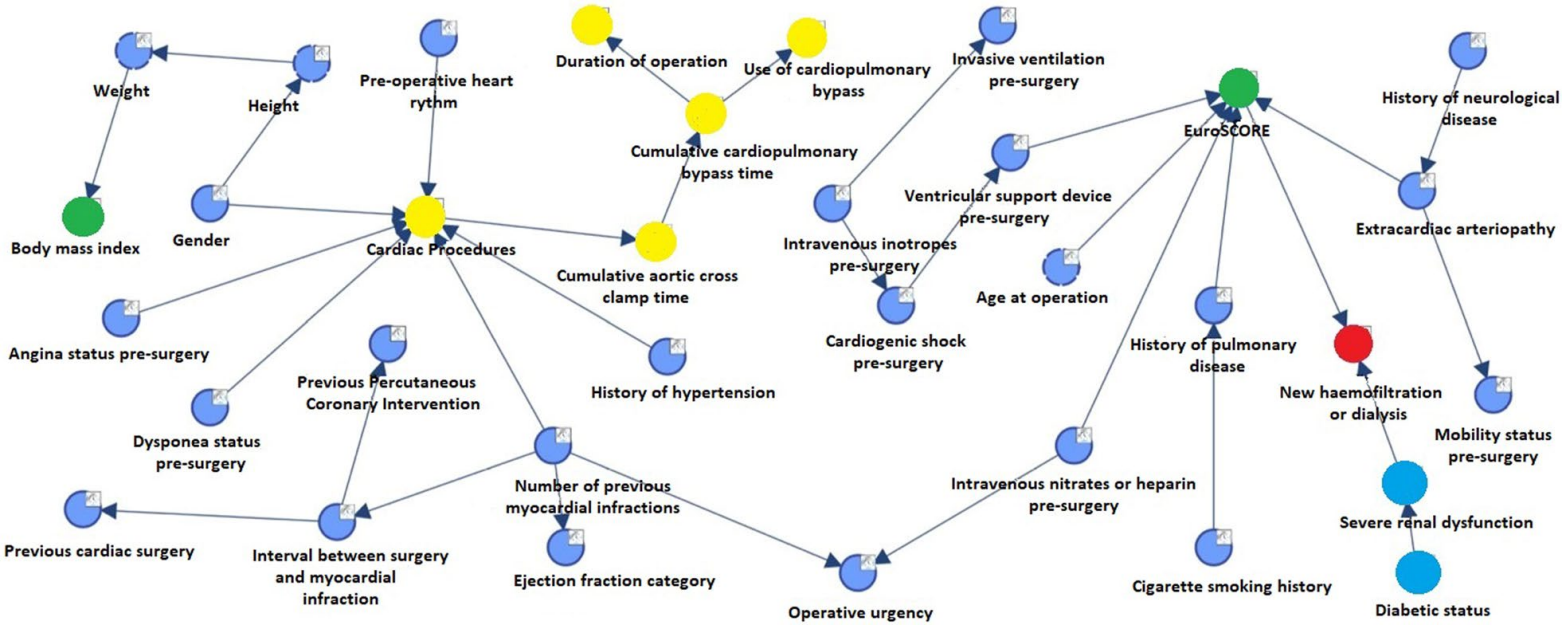
Bayesian network graph with MB for the variable ‘new haemofiltration or dialysis’

‘New haemofiltration or dialysis’ (Fig. 4) was directly related to pre-operative severe renal dysfunction (as determined by creatinine clearance) and ES. Patients with an ES < 2.85 in the absence of severe renal dysfunction had a 1.3% probability of requiring post-operative HF. This rose to 7.4% if the ES was > 8.1. Presence of hypertension and DOO/CPB time did not directly, influence renal outcome.

## Discussion

In the present study, we applied Bayesian networks to investigate principle determinant relationships among pre/peri-operative factors and outcomes in adults undergoing cardiac surgery. Our use of machine learning techniques simultaneously illustrates many of the previously established relationships and offers a number of new ones. This includes establishing the importance of intra-operative variables such as DOO and CPB times to detect/predict adverse outcomes. Indeed, what matters is not exclusively the pre-operative morbidity of the patient.

Our results showed that DOO is linked to survival. Interestingly, ES was not associated with survival in this study as either a parent node or co-dependent of the parent node. In agreement with earlier studies, we show that risk of mortality increases significantly alongside CPB and operation times, as they are inevitably associated with a higher burden of myocardial injury and systemic inflammation despite judicious use of cardioplegia [4, 13]. Notably, Fig. 1 showed that mortality was independent of CPB use given duration of operation. That is to say if the duration of operation was known, the CPB time (by itself) was of no consequence in determining the probability of survival status. Although this does not negate the role CPB and AXC times play, it does invite the question of how prolonged anaesthesia affects myocardial function in the absence of CPB. In this vein, previous authors [14, 15] have suggested that surgical times under general anaesthesia, greater than 180–220 min were a risk factor associated with peri-operative cardiopulmonary complications or death. Studies have compared use of volatile and non-volatile anaesthetic agents on surgical outcomes for OPCAB procedures but not on duration of anaesthesia itself [16, 17]. None the less, long operative times may have had unforeseen technical challenges and a weakness of any predictive model (both Bayes and classic regression) would be its inability to differentiate between planned and unplanned processes of care, e.g., the length of an operation such as aortic valve replacement with synchronous 4 × coronary artery bypass grafts Vs coronary artery bypass grafts × 2 encountering technical complications.

Incidence of stroke and other neurological complications was principally associated with pre-operative inotrope use and unexpectedly had no direct relation to duration of operation or previous history of neurological disease or hypertension. This result should be interpreted with caution, as these factors may very well play a role. In this instance, the theory of Bayesian networks determines that if these factors are already known, then the use of pre-operative inotropic support is a stronger determinant of neurological outcome in our network. However, the ultimate reason for this association in our study remains unclear. Intriguingly, Almassi et al. [18] also noted the peri-operative use of inotropes as an independent risk factor for post-operative stroke (OR 1.55; CI 1.06–2.26; *p* 0.023). Similarly, a study of 11,825 patients undergoing CABG by Likosky et al. [19] found a five-fold increase of stroke rate associated with prolonged use of inotropes. We speculate that patients requiring pre-operative and intra-operative inotropes are pre-disposed to hypotensive episodes as a result of diminished cardiac output thereby triggering cerebral hypoxia.

Re-exploration for bleeding/cardiac tamponade was three times higher for CPB times > 212 min Vs < 99 min. Longer CPB times would encounter greater coagulopathy. One also must consider that longer procedures may also mean technically more challenging cases. Our findings reflect the works of both Moulton [20] and Vivacqua [21] who found CPB time to be an independent predictor of need for re-operation. However, this does discount the OPCAB cases in our cohort, one possible explanation is due to the low number, any incidence of re-exploration for bleeding in this cohort may have been deemed insignificant by the machine.

Our study has shown that although ES cannot be linked to mortality in our data, it predicted new renal replacement therapy, thereby endorsing previous studies. Both Chen et al. [22] and Toumpoulis et al. [23] have shown that ES has good discriminatory ability in predicting post-operative renal failure. Biancari et al. [24] in a study of over 1000 patients showed that ES was good at predicting post-operative renal failure requiring dialysis as well as post-operative length of stay. Also like our study, Muralidhar et al. [25] found pre-operative renal impairment to be a strong predictor of HF. It is likely that such patients have chronically reduced glomerular perfusion pressure which is then augmented by surgery and cardiopulmonary bypass.

It is reassuring that machine generated outcomes in cardiac surgery can confirm established principle determinant associations as well as uncover potential new areas of research. The application of artificial intelligence, in this case of a Bayesian network, to medical decision making has been adopted in several branches of medicine but has so far remained unusual in cardiac surgery [26, 27]. Such methods can be used to form their own predictive models [28]. While this is beyond the scope of this paper, we have identified the variables ‘Duration of Operation’ and ‘CPB Time’ as having significant impact on several post-operative outcomes.

We hope identification of such variables including intra-operative factors can be used in future studies to develop algorithms to predict not only mortality but also morbidity outcomes for adult cardiac surgery. Previous studies have shown CPB duration to be a very strong independent predictor of post-operative morbidity and mortality [4], yet current popular risk stratification tools in cardiac surgery do not use intra-operative variables to calculate outcomes. We believe a future risk stratification model which utilises preoperative as well as perioperative variables (which may be static in their nature but derived from identification of the most significant dynamic factor) would generate a more refined and accurate risk tool. Indeed, the variable pool will be enlarged and thus one would be more likely to create and validate predictive models with superior measures of predictive performance such as accuracy upon rigorous cross validation procedures. Finally, these models would have the advantage of being based on principle determinant pathways which is highly relevant in clinical applicability [29]. Use of intra-operative variables is not a novel concept in surgery [30] but remains elusive from ES and the STS risk calculator.

Several outcomes of interest such as use of post operative blood products, inotropic and mechanical ventricular support were not considered primary clinical outcomes due to the subjective variability of planned/unplanned processes of care. The MB for these outcomes is included in the Additional file 1. Derivation of an evolved risk stratification tool therefore would limit its applicability only to definitive endpoints of care (e.g., death, stroke, renal failure). Application to other outcomes such as Deep Sternal Wound Infection (DSWI), would require standardised protocol management, a larger study population and longer follow up.

However, our study has shown a proof of concept that Bayesian networks created via machine learning can be used to identify relevant determinants of short-term outcomes in adult cardiac surgery. It is our hope the information presented in this paper will stimulate debate for others to consider the importance of both pre- and intra-operative factors for future risk stratification models of adult cardiac surgery (Additional file 2: Figure S1, Additional file 3: Figure S2, Additional file 4: Figure S3, Additional file 5: Figure S4).

### Limitations

Our study is a single-institutional retrospective study, which limits the generalizability of results. The reported morbidity is in-patient only and we do not have longer term follow up data. The number of deaths, CVA and DSWI that were observed were very small and a larger study population may infer different results. Cause of death is unknown and therefore does not allow further scrutiny. Our cohort had heterogeneous operations and so our results are difficult to interpret for specific operations or practical applications. However, our results indicate that operative and CPB times were the most influential factor on several outcomes despite the nature of operation. Data on any technical issues encountered during a procedure, which may have lengthened CPB, and AXC time is lacking and could have a high association with the negative outcomes that influence Bayesian modelling. We do not have data on several clinically relevant variables, which may have influenced our results. Furthermore, our data is confounded because of insufficient detail in the data sample, for example, our analysis does not measure the mode of myocardial protection for each procedure: in our study, there was an amalgamation of OPCAB; cold antegrade/retrograde blood cardioplegia (± ‘hotshot’ technique) and intermittent cross clamp fibrillation. In the absence of randomized data, causality cannot be inferred from our MB alone. Finally, our study of pre-operative variables was focused on those routinely captured to calculate ES and ignored other significant pre-operative factors such as frailty status [31] and serum albumin concentration [32].

## Conclusions

In summary, we present the first use of machine learning Bayesian networks to determine outcomes of interests in adult cardiac surgery in a UK population. We found intra-operative factors, were the most prominent variables associated with survival and return to theatre for bleeding. We hope that use of such machine learning techniques can be used in larger samples to develop predictive risk models in the future.

## Supplementary Information

**Additional file 1.** Supplementary Materials.

**Additional file 2.** **Figure S1.** Bayesian Network Graph with MB for the variable ‘Post-operative red blood cell transfusion’.

**Additional file 3.** **Figure S2.** Bayesian Network Graph with MB for the variable ‘post-operative ventricular support’.

**Additional file 4.** **Figure S3.** Bayesian Network Graph with MB for the variable ‘post-operative length of stay’.

**Additional file 5.** **Figure S4.** Bayesian Network Graph with MB for the variable ‘Deep Sternal Wound Infection’.

## Data Availability

The datasets used and/or analysed during the current study are available from the corresponding author on reasonable request.
