# Supplementary material for: Bayesian networks identify determinants of outcomes following cardiac surgery in a UK population
Source: BMC Cardiovasc Disord. 2023 Feb 6;23:70. doi: 10.1186/s12872-023-03100-6 (PMC9903419; doi:10.1186/s12872-023-03100-6)
Supplement: Supplementary file 1 — Additional file 1. Supplementary Materials. [file 12872_2023_3100_MOESM1_ESM.docx]

**Additional file 1**

Extended Statistical Methodology

We used the machinery of Bayesian networks to identify the smallest subset of variables that could provide the necessary information for prediction of the outcomes of interest consistent with known, or possibly newly discovered logically consistent principle determinant relationships between the variables. Classical statistics methodology is inappropriate for this purpose (1). Indeed, the study demonstrates the ability of linear regression and other higher order models to accurately predict variables of interest even if there are neither logical nor principle determinant relationships between the independent variables and the corresponding dependent variable. A Bayesian network is a probabilistic directed acyclic graphical model used to estimate and visualise the inter-dependencies and principle determinant relationships among many variables (2). The model uses a graph where each variable is represented by a ‘node’ and the variables are linked by arrows to represent interdependencies. It is possible to quickly visualise dependencies using the concept of a “Markov blanket” (MB). The variable E is said to be a parent of A if there is an arrow emerging from node E and pointing to node A, i.e., E→A, equivalently, A is a child of E. The Markov blanket of a node A is the set of its parents, its children and its children’s other parents (co-parents). A fundamental property of Bayesian networks states that a variable ‘A’ is independent of any other variable (‘B’), given its MB. This means that the MB contains all the variables necessary to predict A. For example, from Figure 1, the variable ‘History of pulmonary disease’ has the variable ‘cigarette smoking history’ as its only ‘parent’ while it has one ‘child’: ’EuroSCORE’.

Bayesian networks are defined for categorical data; therefore, prior to any network learning, all continuous variables must be discretised. This is achieved via ‘k-means’ clustering algorithms. More specifically, *k*-means clustering is a method of [vector quantization](https://en.wikipedia.org/wiki/Vector_quantization) that aims to [partition](https://en.wikipedia.org/wiki/Partition_of_a_set) *m* empirical observations into *k* clusters such that each observation belongs to the [cluster](https://en.wikipedia.org/wiki/Cluster_(statistics)) with the nearest [mean](https://en.wikipedia.org/wiki/Mean) while minimizing the within-cluster variances (3). We undertook this approach, as opposed to human-specified cut-offs, for the reasons of consistency as, to the best of our knowledge, there is no scientific consensus regarding the universality of these cut-offs for all eight continuous explanatory variables. Details of discretisation cut-offs for other variables are presented in Tables S1-S8 in the Supplementary Material. The search space for the network-learning algorithm was reduced by mandating that future events cannot be a principle determinant of past observations (4). This was achieved by assigning temporal values to all nodes. All baseline variables were assigned a temporal index of 1. ES and Body Mass Index were assigned a temporal index of 2 as they can only be computed once the corresponding baseline variables that define them are collected. All intra-operative variables were assigned a temporal index of 3, while all the outcomes of interest were assigned a temporal index of 4. Details of discretisation categorical outcomes and all variable temporal values are provided in Table S9. Unsupervised machine learning techniques were subsequently used to learn the data’s principle determinant structure for each primary outcome on the reduced search space emerging from the temporal causality constraint. In other words, the variables with larger temporal indices were prohibited from being parental nodes of variables with a smaller temporal index.

Table S1. Survival discretisation categories: Markov blanket consists only of the variable ‘’Duration of Operation”. The corresponding conditional probability table is given below:

| Duration of operation (minutes) | Alive | Dead |
| --- | --- | --- |
| <245 | 99.05 | 0.95 |
| >245 and ≤ 421 | 98.51 | 1.49 |
| >421 | 88.18 | 11.82 |

Table S2. Neurological deficit discretisation categories: Markov blanket consists only of the variable ‘’Intravenous inotropes pre-surgery”. The corresponding conditional probability table is given below:

| Intravenous inotropes prior to surgery | CVA/TIA/Other | No |
| --- | --- | --- |
| No | 0.99 | 99.01 |
| Yes | 20.00 | 80.00 |

Table S3. Return to theatre for bleeding / tamponade discretisation categories: Markov blanket consists only of the variable ‘’Cumulative cardiopulmonary bypass time”. The corresponding conditional probability table is given below:

| Cumulative cardiopulmonary bypass time (minutes) | No | Yes |
| --- | --- | --- |
| ≤ 99 | 96.96 | 3.04 |
| >99, ≤ 212 | 93.66 | 6.34 |
| >212 | 90.61 | 9.39 |

Table S4. New Haemofiltration/dialysis discretisation categories: Markov blanket consists of the variables ‘’EuroSCORE” and ‘’Severe renal dysfunction”. The corresponding conditional probability table is given below:

| EuroSCORE | Severe renal dysfunction | No | Yes |
| --- | --- | --- | --- |
| ≤2.85 | Acute failure ≤ 6 weeks prior to surgery | 100.00 | 0.00 |
|  | Chronic failure > 6 weeks before surgery | 90.91 | 9.09 |
|  | No dialysis but anuria or oliguria | 80.00 | 20.00 |
|  | None | 98.74 | 1.26 |
| >2.85 and ≤ 8.11 | Acute failure ≤ 6 weeks prior to surgery | 66.67 | 33.33 |
|  | Chronic failure > 6 weeks before surgery | 75.00 | 25.00 |
|  | No dialysis but anuria or oliguria | 100.00 | 0.000 |
|  | None | 95.11 | 4.89 |
| >8.11 | Acute failure ≤ 6 weeks prior to surgery | 100.00 | 0.00 |
|  | Chronic failure > 6 weeks before surgery | 66.67 | 33.33 |
|  | No dialysis but anuria or oliguria | 100.00 | 0.00 |
|  | None | 92.57 | 7.43 |

Table S5. Post-operative Red Blood Cell transfusion discretisation categories: Markov blanket consists only of the variable ‘’Cumulative bypass time”. The corresponding conditional probability table is given below:

| Cumulative cardiopulmonary bypass time (minutes) | No | Yes |
| --- | --- | --- |
| ≤99 | 72.09 | 27.91 |
| >99 and ≤ 212 | 55.27 | 44.73 |
| >212 | 42.72 | 57.28 |

Table S6. Post-operative inotrope / mechanical ventricular support discretisation categories: Markov blanket consists only of the variable ‘’EuroSCORE”. The corresponding conditional probability table is given below:

| EuroSCORE | Inotropes | Inotropes & Ventricular assist device | No support | Ventricular assist device only |
| --- | --- | --- | --- | --- |
| ≤2.85 | 27.26 | 0.31 | 72.23 | 0.20 |
| >2.85 and ≤ 8.11 | 40.72 | 0.09 | 59.10 | 0.09 |
| >8.11 | 48.82 | 0.95 | 49.76 | 0.47 |

Table S7. Length of post-operative stay discretisation categories: Markov blanket consists only of the variable ‘’EuroSCORE”. The corresponding conditional probability table is given below:

| EuroSCORE | ≤13 days | >13and ≤37 days | >37days |
| --- | --- | --- | --- |
| ≤2.85 | 91.15 | 8.16 | 0.69 |
| >2.85 and ≤8.11 | 73.52 | 21.96 | 4.52 |
| >8.11 | 64.93 | 27.96 | 7.11 |

Table S8. Deep sternal wound infection discretisation categories: Markov blanket consists only of the variable ‘’Diabetes management”. The corresponding conditional probability table is given below:

| Diabetes status | No DSWI | DSWI |
| --- | --- | --- |
| Diet controlled | 98.50 | 1.50 |
| Insulin | 97.15 | 2.85 |
| Not diabetic | 99.22 | 0.78 |
| Oral therapy | 99.42 | 0.58 |

Use of homologous red blood cells post-operatively was dependent upon CPB time (Figure 5 in supplementary material). In those with CPB times <99.3 minutes, RBC transfusion was necessary in 28% of cases – in those with CBP times >212 minutes, it was 57%. ES was found to be the strongest influence in determining post-operative inotrope/mechanical ventricular support (Figure 6 in supplementary material) as well as length of post-operative stay (Figure 7 in supplementary material). Seventy-two percent of low-risk patients (ES <2.85) did not require post-operative inotropic/mechanical support, for high-risk patients (ES >8.11) this occurred in only 49.8% of cases. Post-operative stays under 13 days were achieved for 91.2% of low-risk patients and 64.9% of high-risk patients. DSWI was linked to diabetic status (Figure 8 in supplementary index):

Table S9. Shows the temporal value assigned for each variable prior to unsupervised machine learning algorithms to generate Markov blankets of interest.

| **Variable** | **Temporal Value** |
| --- | --- |
| Gender | 1 |
| Age at operation | 1 |
| Angina status | 1 |
| Dyspnoea status | 1 |
| Number of previous myocardial infarctions | 1 |
| Interval between surgery and myocardial infarction | 1 |
| Previous percutaneous coronary intervention | 1 |
| Previous cardiac surgery | 1 |
| Diabetic status | 1 |
| Cigarette smoking history | 1 |
| Hypertension | 1 |
| Severe renal dysfunction | 1 |
| History of pulmonary disease | 1 |
| History of neurological disease | 1 |
| Extra-cardiac arteriopathy | 1 |
| Pre-operative heart rhythm | 1 |
| Left ventricle ejection fraction category | 1 |
| Intravenous inotropes/ nitrates/ heparin pre-surgery | 1 |
| Invasive ventilation pre-surgery | 1 |
| Cardiogenic shock pre-surgery | 1 |
| Ventricular support device pre-surgery | 1 |
| Operative urgency | 1 |
| Cardiac procedure | 3 |
| Use of cardiopulmonary bypass | 3 |
| Return to theatre for bleeding / tamponade | 4 |
| Post-operative inotropic / mechanical ventricular support | 4 |
| Post-operative arrythmia | 4 |
| Red blood cell transfusion | 4 |
| Deep sternal wound infection | 4 |
| New neurological deficit | 4 |
| New haemofiltration / dialysis | 4 |
| Patient status at discharge | 4 |
| Duration of operation | 3 |
| Post-operative stay in days | 4 |
| Body Mass Index | 2 |
| EuroSCORE | 2 |
| Height (cm) | 1 |
| Weight (kg) | 1 |
| Mobility status | 1 |
| Cumulative cardiopulmonary bypass time | 3 |
| Cumulative aortic cross clamp time | 3 |

**Additional figures**

Figure S1. Bayesian Network Graph with MB for the variable ‘Post-operative red blood cell transfusion’

Figure S2. Bayesian Network Graph with MB for the variable ‘post-operative ventricular support’

Figure S3. Bayesian Network Graph with MB for the variable ‘post-operative length of stay’

Figure S4. Bayesian Network Graph with MB for the variable ‘Deep Sternal Wound Infection’

**References**

1. Stupid Data Miner Tricks: Overfitting the S&P 500 data, David J. Leinweber, The Journal of Investing, 16(1); 15-22, January 2007

2. Pearl J. Probabilistic reasoning in intelligent systems: networks of plausible inference. San Mateo, Calif.: Morgan Kaufmann Publishers; 1988. xix, 552 p. p.

3. Steinhaus H. Sur la division des corp materiels en parties. Bulletin Polish Acad Sci Math. 1956; 1:801-4.

4. Explanatory Causal Analysis with Time Series Data, James M. McCracken in Synthesis Lectures on Data Mining and Knowledge Discovery, Series Editors: Jiawen Han, Lise Getoor, Wei Wang, Jahannes Gehrke, Robert Grossaman, Morgan and Claypool Publishers, 2016.
