# Supplementary figures and images for: Bayesian networks identify determinants of outcomes following cardiac surgery in a UK population
Source: BMC Cardiovasc Disord. 2023 Feb 6;23:70. doi: 10.1186/s12872-023-03100-6 (PMC9903419; doi:10.1186/s12872-023-03100-6)

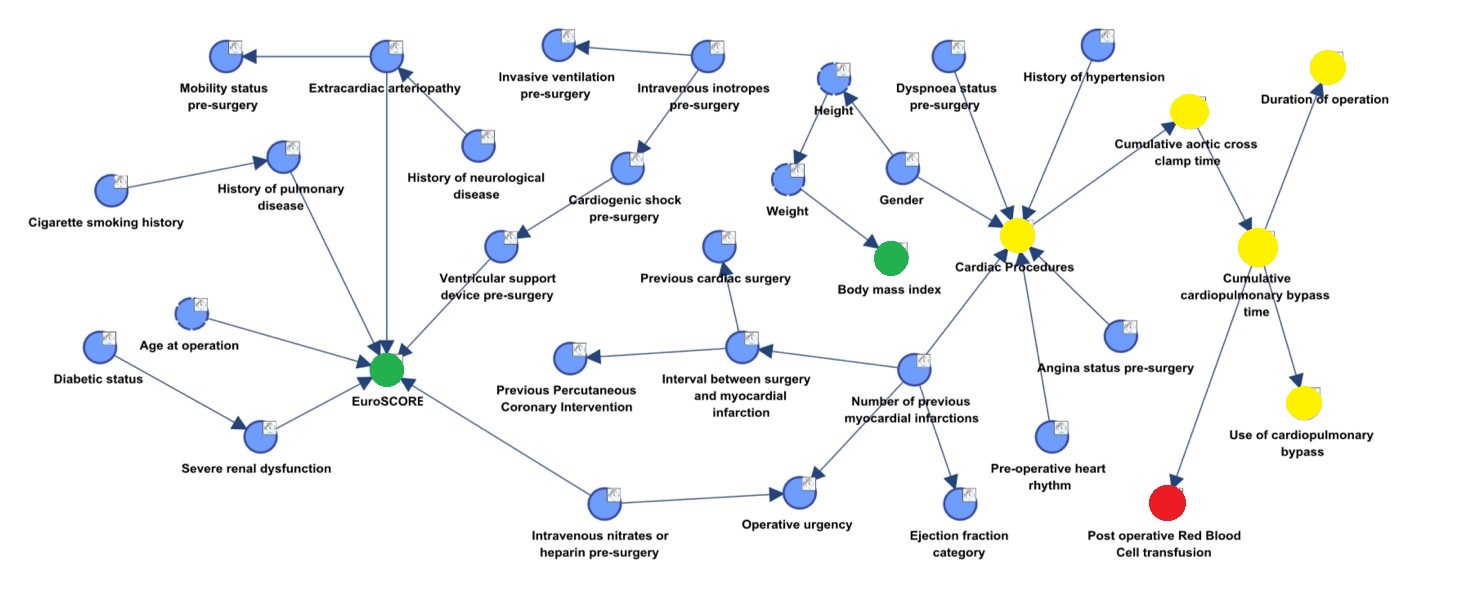

Supplement: Supplementary file 2 — Additional file 2. Figure S1. Bayesian Network Graph with MB for the variable ‘Post-operative red blood cell transfusion’. [file 12872_2023_3100_MOESM2_ESM.jpg]

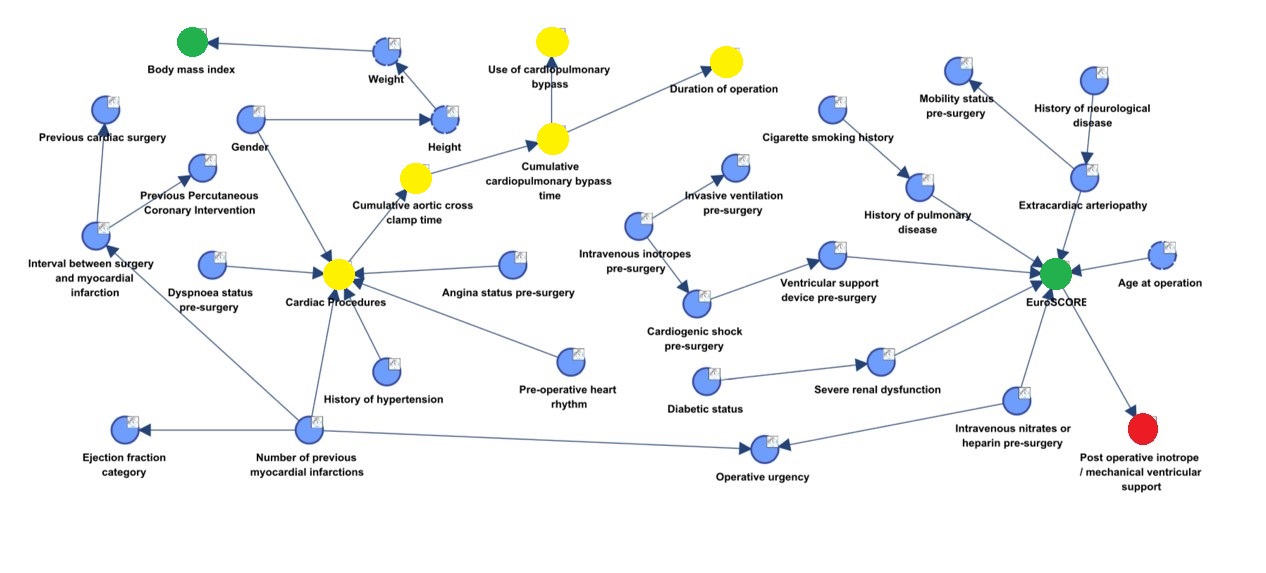

Supplement: Supplementary file 3 — Additional file 3. Figure S2. Bayesian Network Graph with MB for the variable ‘post-operative ventricular support’. [file 12872_2023_3100_MOESM3_ESM.jpg]

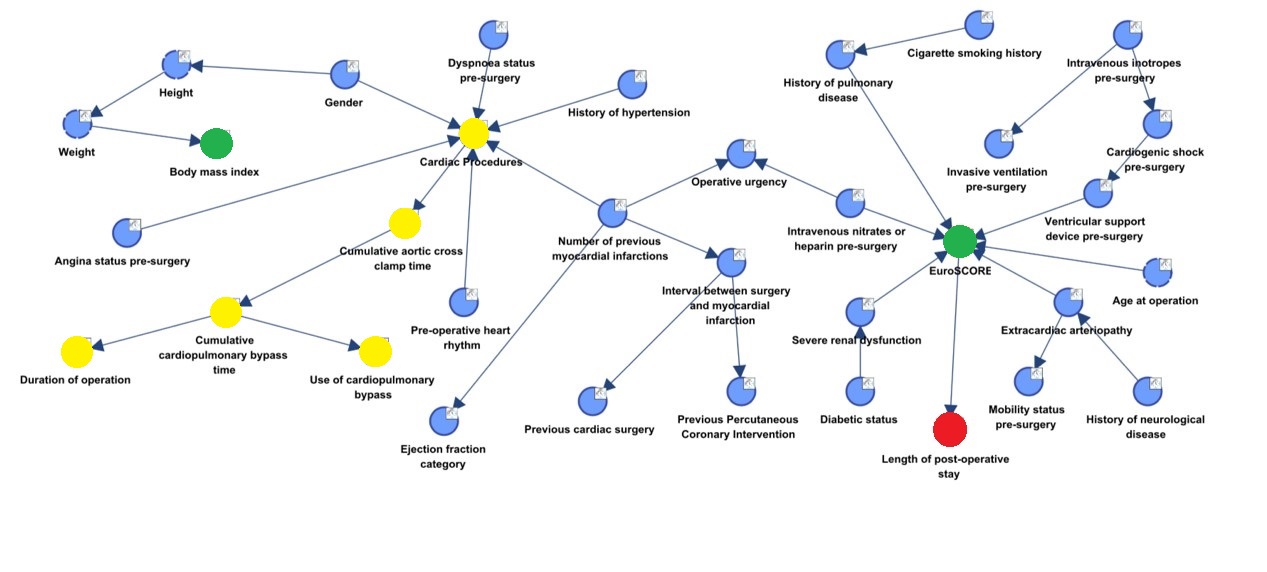

Supplement: Supplementary file 4 — Additional file 4. Figure S3. Bayesian Network Graph with MB for the variable ‘post-operative length of stay’. [file 12872_2023_3100_MOESM4_ESM.jpg]

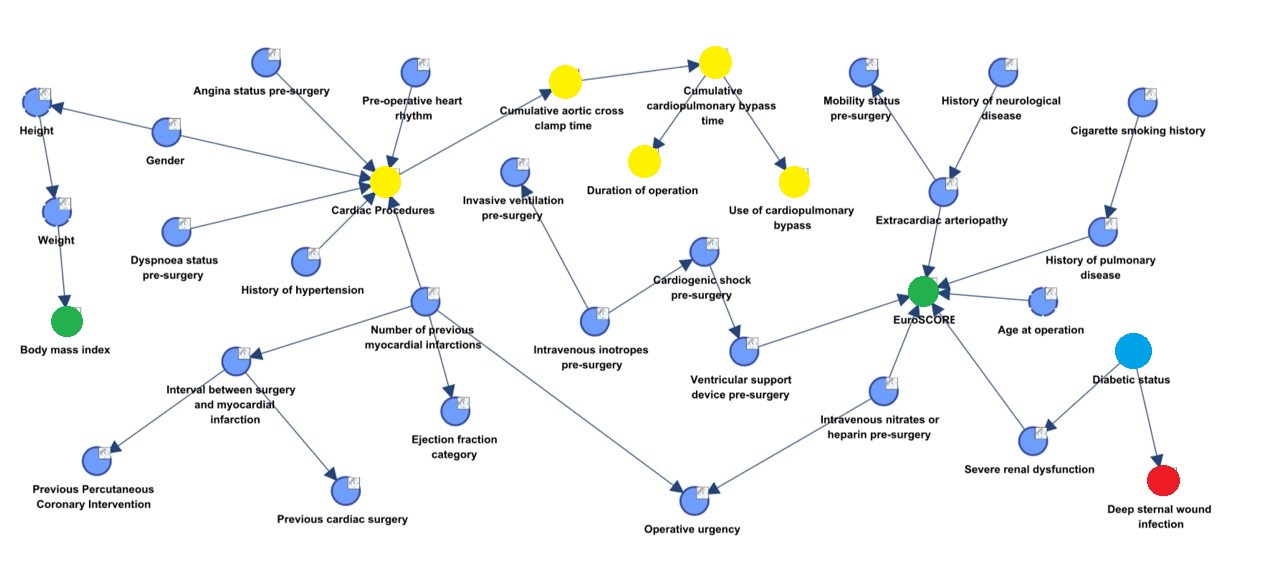

Supplement: Supplementary file 5 — Additional file 5. Figure S4. Bayesian Network Graph with MB for the variable ‘Deep Sternal Wound Infection’. [file 12872_2023_3100_MOESM5_ESM.jpg]
